# Considerations for Health Researchers Using Social Media for Knowledge Translation: Multiple Case Study

**DOI:** 10.2196/15121

**Published:** 2020-07-23

**Authors:** Sarah A Elliott, Michele P Dyson, Gilbert V Wilkes, Gabrielle L Zimmermann, Christine T Chambers, Kristy DM Wittmeier, Dianne J Russell, Shannon D Scott, Denise Thomson, Lisa Hartling

**Affiliations:** 1 Alberta Research Center for Health Evidence Department of Pediatrics University of Alberta Edmonton, AB Canada; 2 Alberta SPOR SUPPORT Unit Department of Pediatrics University of Alberta Edmonton, AB Canada; 3 Cochrane Child Health Edmonton, AB Canada; 4 School of Communication Studies Mount Royal University Calgary, AB Canada; 5 Department of Community Health Sciences University of Calgary Calgary, AB Canada; 6 Departments of Psychology & Neuroscience and Pediatrics Dalhousie University Halifax, NS Canada; 7 Centre for Pediatrics Pain Research IWK Health Centre Hallifax, NS Canada; 8 Department of Pediatrics and Child Health Rady Faculty of Health Sciences University of Manitoba Winnipeg, MB Canada; 9 Children’s Hospital Research Institute of Manitoba Winnipeg, MB Canada; 10 CanChild Centre for Childhood Disability Research McMaster University Hamilton, ON Canada; 11 Faculty of Nursing University of Alberta Edmonton, AB Canada

**Keywords:** social media, knowledge translation, health research, engagement, dissemination, exchange, evaluation

## Abstract

**Background:**

Despite extensive literature describing the use of social media in health research, a gap exists around best practices in establishing, implementing, and evaluating an effective social media knowledge translation (KT) and exchange strategies.

**Objective:**

This study aims to examine successes, challenges, and lessons learned from using social media within health research and to create practical considerations to guide other researchers.

**Methods:**

The Knowledge Translation Platform of the Alberta Strategy for Patient-Oriented Research SUPPORT Unit formed a national working group involving platform staff, academics, and a parent representative with experience using social media for health research. We collected and analyzed 4 case studies that used a variety of social media platforms and evaluation methods. The case studies covered a spectrum of initiatives from participant recruitment and data collection to dissemination, engagement, and evaluation. Methods and findings from each case study as well as barriers and facilitators encountered were summarized. Through iterative discussions, we converged on recommendations and considerations for health researchers planning to use social media for KT.

**Results:**

We provide recommendations for elements to consider when developing a social media KT strategy: (1) set a clear goal and identify a theory, framework, or model that aligns with the project goals and objectives; (2) understand the intended audience (use social network mapping to learn what platforms and social influences are available); (3) choose a platform or platforms that meet the needs of the intended audience and align well with the research team’s capabilities (can you tap into an existing network, and what mode of communication does it support?); (4) tailor messages to meet user needs and platform requirements (eg, plain language and word restrictions); (5) consider timing, frequency, and duration of messaging as well as the nature of interactions (ie, social filtering and negotiated awareness); (6) ensure adequate resources and personnel are available (eg, content creators, project coordinators, communications experts, and audience stakeholder or patient advocate); (7) develop an evaluation plan a priori driven by goals and types of data available (ie, quantitative and qualitative); and (8) consider ethical approvals needed (driven by evaluation and type of data collection).

**Conclusions:**

In the absence of a comprehensive framework to guide health researchers using social media for KT, we provide several key considerations. Future research will help validate the proposed components and create a body of evidence around best practices for using and evaluating social media as part of a KT strategy

## Introduction

### Background

The concept of social media has been constantly evolving alongside advances in technology and the development and abandonment of various platforms. Social media has now become an integral means for sharing information while engaging and interacting with others across all sectors of society [1,2]. Subsequently, engaging with anyone, anywhere, at any time has presented opportunities for people, businesses, nonprofit organizations, and health care organizations to engage with a wide audience [3,4]. Social media have changed people’s relationships with how they find and use information. Health care consumers (ie, members of the public) can now use web-based platforms to search for health information, seek others’ experiences and opinions, create their own interest and advocacy groups to raise awareness, fundraise, and share information among like-minded communities. Similarly, health researchers have started to capitalize on social media for a number of purposes, from patient engagement to research dissemination and exchange, with countless applications in between [5-7]. Evidence suggests that health researchers largely hold favorable views with respect to the use of social media in a professional capacity [8,9], considering it to provide an engaging and convenient source of information [10], while meeting their need to gain and exchange knowledge with relevant stakeholders [6]. Despite the enthusiasm, there remains a gap around best practices in developing and implementing social media as part of a successful knowledge translation (KT) strategy.

### Knowledge Translation Theories and Social Media Models

Many KT strategies (often guided by theories, models, or frameworks) require that the goals, audience, and messaging be carefully considered to ensure success. However, there is no current KT theory, model, or framework that addresses what makes a successful *social media* KT strategy. The unique requirements that social media has around the what (materials and procedures needed), who (provider), and how (mode of delivery and platform and to whom) are quite nuanced. Likewise, current social networking theories and models do not adequately address all facets of dissemination, exchange, and application of knowledge needed to develop (and evaluate) a KT strategy [2,11-13].

Integrated KT models emphasize that translation of knowledge is expedited when knowledge producers (eg, researchers and scientists) and knowledge users (eg, patients, caregivers, and policy makers) are known to one another and are familiar with one another’s needs, preferences, and circumstances [14,15]. Pick et al [16] conceptualized the 4 Cs of social media in an attempt to describe how social media is used, proposing that *content* is developed within a given *context*, to make *connections* leading to *conversations*. However, the ability of social media users to successfully share, mobilize, and cocreate knowledge still needs to be understood.

### Connecting the Dots: Social Media and Knowledge Translation

We propose that to facilitate effective use of social media as part of a KT strategy, researchers need to know how to reach and engage their target audience through social media, with the information the research teams want to share. Likewise, considerations around what stakeholders want to hear and learn need to be incorporated. There are 3 components of social media that can be classified as explanatory and predictive, which could help guide this [17]:

1. Posting frequency, which means how often a user (person, group, or enterprise) circulates content, asks for input, or responds to a comment or request, without respect to any other factor, that is, quality, depth of detail (whether a post is a simple headline or a detailed infographic), and kind of post (response to another user, announcement of an event or a resource, or key findings of a study) [17-19].
2. Whether and to what degree the user can affect or create a sense of awareness (or negotiated awareness) of who they are, their purpose, their location, the character of their expertise or experience, and the precise services or information they have on hand for their intended audience [17].
3. Whether and to what degree the user allows or affords other users to select effectively within their content and to find what they need to find and whether and to what degree the selection activity is used by the user as grounds to optimize or refine their offerings (social filtering) [20].

To date, no KT or social media framework incorporates these explanatory and predictive components. In the absence of a suitable framework, we draw on 4 case studies using social media for engagement and dissemination of health research. We summarize the successes, challenges, and lessons learned and present recommendations for others considering social media as part of a KT strategy.

## Methods

### General Approach

In 2016, the Alberta Strategy for Patient-Oriented Research (SPOR) SUPPORT Unit’s Knowledge Translation Platform hosted a national meeting (which included a number of KT and communication experts, platform staff, and consumers) to explore the potential linkage between social media and KT research. Subsequently, a national working group was formed of representatives (n=12) from academia (ie, faculty and research staff) and community (ie, knowledge brokers and parent representative), all with experience using social media as part of a KT strategy for health research. The working group defined the approach to the topic and scope of the project. The goal was to create a document that compared researchers’ experiences and provided considerations to guide other researchers wanting to utilize social media as part of a KT strategy. The working group met monthly over the course of a year (November 2017 to November 2018), collectively identified possible case studies for inclusion based on pre-established inclusion criteria (described below), and developed data collection forms for extracting key findings. Once data from each case study were collected and summarized, deliberative dialogue [21] was used to draw out important concepts and points to consider for discussion. Working group members were then responsible for creating considerations for other researchers through iterative discussions. All working group members were invited to help draft the final report and be part of the authors’ team.

### Social Media Case Studies

The aim was to collect case studies of social media use in health research that covered a spectrum of KT initiatives as well as a mix of social media platforms and evaluation methods. Case studies had to be focused on child health, include an evaluation component, and have been published in the past 5 years. The working group (n=12) first looked internally to gather case studies and evaluated the collective breadth of experience using social media for KT activities. An invitation was then extended to other research groups to supplement the working group’s experiences. Researchers providing case studies were also invited to be part of the working group and authors’ team. Four case studies were selected by the working group for inclusion. The authors of the case studies were then asked to provide information and data about the project in a pre-established data collection form. The data collection form gathered information about the case study’s topic and objectives, intended audience, project logistics (eg, social media platform(s) used, staffing requirements, and project timeline), use of any predictive or explanatory factors such as posting frequency, negotiated awareness and social filtering, dimensions of communication used (Multimedia Appendix 1), evaluation methods (including outcomes and metrics), key findings, and self-reported lessons learned (ie, successes and challenges). To ensure validity and reliability of the data collected from the case studies, collated and summarized data were presented to case study authors for review.

### Group Discussions and Development of Considerations

Once data were collected, a summary document that highlighted the similarities and differences between the case studies was created. Self-reported barriers and facilitators to KT encountered when using social media were also collated. Barriers and facilitators were coded into categories based on what part of the KT strategy they impacted (eg, planning, doing, and evaluating). These categories were then broken down into specific elements that formed part of each category (eg, planning—personnel and resource requirements, platform used, and scope of messaging). This prompted focused discussions on the overall key successes and challenges. Working from the summary tables and coded elements, deliberative dialogue [21] (facilitated by SE) was used to draw out important concepts and points to consider for discussion. Through an iterative discussion process, the working group converged on considerations for researchers planning to use social media for KT, guided by the explanatory and predictive components of social media.

## Results

### Summary of Case Studies

The 4 case studies are summarized in Table 1. More details are presented in Multimedia Appendix 2.

Here, we synthesize the common case study elements (processes of implementing the social media KT strategies) with regard to lessons learned and challenges faced.

**Table 1:** Case study summaries.

Case study	Objective	Intended audience	Platform	Dimensions of communication^a^	Time frame and intensity (posting frequency)	Social filtering	Negotiated awareness	Staffing requirements	Evaluation
Cochrane Child Health [7]	Dissemination	Child health HCP^b^, child health researchers, and health care organizations	Twitter and blog	Asynchronous, one to many, dynamic, pull, remote, and iconic indexical	6 months, weekly blog posts, daily tweets, monthly journal club on Twitter	Influencers were tagged in tweets; hashtags were used to identify conditions under discussion	Content shared by influencers tagged and research group stakeholders	0.2 FTE^c^ RC^d^, 0.2 FTE, information specialist, and 0.3 FTE graduate student or RA^e^	Twitter analytics, Bitly statistics, Altmetric scores, journal club, and participant survey feedback
#ItDoesntHaveToHurt [22]	Dissemination and engagement	Parents (primarily mothers)	Blog, Twitter, Facebook, Instagram, YouTube, and stakeholder websites	Asynchronous, one to many, dynamic, remote, recorded, focal, push, durable, phonetic syllabic, and iconic indexical	12 months, varied with approximately 3 posts per month plus amplification and sharing	Content appeared on partner website and social media with a well-established community of parents, influencer promotion and engagement (parent bloggers across the country amplified content using their own social channels), hashtag use #ItDoesntHaveToHurt	Content shared by influencers, stakeholders, research groups’ social media channels, and research partners’ social media channels	0.75 FTE RC, 0.2 FTE, stakeholder PC^f^, and digital content creators, as needed	Web-based analytics, pre- and postsurveys and interviews, social listening and sentiment analysis, media analysis, partnership analysis, and patient engagement evaluation
Hirschsprung’s Disease Community [23]	Engagement (with caregivers to identify knowledge gaps) and knowledge exchange	Caregivers of children with Hirschsprung disease	Twitter, Facebook, and blog	Asynchronous, many to one, dynamic, and remote	1-month, daily interaction from parent-led administrative team, weekly posting and reminders during research	Community was pre-established	Posts and messages were from the administrator of an established community; the community has a clear focus, consistent posting, and committed membership	0.5 FTE RA, parent partner (ongoing community management), and communication staff (ongoing community management)	Google analytics and number of surveys completed
Parents Participating in Research Facebook Group [24]	Knowledge exchange and dialogue	Parents and caregivers of special needs children and child health researchers	Facebook	Asynchronous, many to many, and synchronous	9 months, varied with approximately 4 posts per week	Parent moderators direct messaged or tagged topics to parents and research contributors with possible perspectives to add to the discussion	Content shared by moderators, parent and caregivers, or researchers	2 parent moderators—approximately 0.4-0.7 FTE; KT^g^ committee and parent moderator liaison—approximately 0.5 FTE	Analytics of engagement and activity (eg, number of posts and number of comments) and web-based survey of members

^a^See Multimedia Appendix 3.

^b^HCP: health care providers.

^c^FTE: full-time equivalent.

^d^RC: research coordinator.

^e^RA: research assistant.

^f^PC: project coordinator.

^g^KT: knowledge translation.

### Planning

It was unanimous that advanced planning was a key component to a successful social media KT strategy. Furthermore, a prior understanding of what theory, model, or framework best suited the goal of the KT strategy was helpful in planning the different approaches. The *Cochrane Child Health* strategy focused on disseminating information. As knowledge producers, they planned and implemented approaches to push (disseminate) knowledge toward audiences who they believed could benefit from receiving it (health care providers and researchers). The *Hirschsprung’s Disease Community* and *#ItDoesntHaveToHurt* employed strategies to build upon pre-established web communities. They leveraged partnerships to foster engagement and expedite information exchange with their intended audience (parents). The *Hirschsprung’s Disease Community*, *#ItDoesntHaveToHurt*, and *Parents Participating in Research Facebook Group* initiatives planned and implemented strategies to gather knowledge from sources (parents and caregivers) they identified as producing knowledge that was useful to their decision making and research agenda (priority setting and knowledge gaps). Additionally, to further build their communities, they planned to engage with their users through an interactive process (exchanging information through discussion boards, question and answer sessions, survey responses, and feedback sessions).

It was also noted that case studies focusing on a specific topic or disease led to engaging a more saturated audience. One case study reported that it was difficult to tap into any specific existing audience when posting about diverse clinical topics, whereas others that had a more focused topic (that meshed well with the focus of the pre-existing group) felt that a tighter community was developed, which enhanced information sharing.

Planning for the required staff and resources to successfully implement and maintain the KT strategy, including frequent posts and audience interactions, was challenging. The time frame from the case studies ranged from 1 month to 1 year. Case study authors reported not knowing how long a strategy needed to be for it to be effective. The main driver for the length of a strategy was the availability of financial and human resources support.

Many case studies made use of programs to help manage their social media campaigns. Tools such as Buffer (Buffer Inc, 2010) were used to preschedule tweets (the bulk of the campaigns), allowing for monitoring and interactions to be completed throughout the campaign. To keep track of this, the case studies used tools such as TweetDeck (Twitter, 2008) or Hootsuite (Hootsuite Inc, 2008), which provided a user-friendly interface to track and monitor the account, as well as log interaction notifications and collect analytics.

### Doing

#### Platforms, Models of Communication, and Posting Frequency

A variety of different platforms were utilized among the case studies to increase reach and engagement. Three of the case studies used a multiplatform approach in which Twitter, Facebook, Instagram, and blogs were used to cross-post information and engage with multiple audiences across an expansive social network. Cochrane Child Health, whose target audience was health care providers, researchers, and organizations, focused on using Twitter and blog posts and hosting web-based journal clubs. Case studies targeting parents and caregivers, however, tended to use Facebook, YouTube, and Instagram in addition to Twitter. This was a strategic element, based on prior work to understand who their audiences were and where they gathered on the web. It was also noted that having an established platform or network (eg, Cochrane Child Health had >3500 followers at the time of the campaign) was easier than building a network from scratch for a specific campaign. Those that had a pre-established web presence or leveraged partners with such a pre-established presence reported greater interactions and reach.

A common element throughout the case studies was the dimension of communication, with all using an asynchronous approach in which messages were exchanged intermittently rather than in a steady stream. The intensity of communication varied greatly from daily to weekly between and within each case study. Across all case studies, we found that engaging the intended audience through specific posts, rather than constantly passively pushing information, amplified the sharing of information between users. However, it was noted that there was more web-based interaction overall with more frequent posts. Posting frequency was also dictated by platform use. On a platform in which information exchange occurs rapidly in real time (eg, Twitter), it was found to be important to post quite frequently; otherwise, users tended to disengage. On other platforms such as Facebook, 1 case study received feedback from their audience, suggesting that the ability for members to be able to read and contribute at any time (day or night) was what kept them engaged. Allowing users to contribute to ongoing community conversations enhanced engagement and exchange practices.

#### Negotiated Awareness, Social Filtering, and Content Development

Negotiated awareness and social filtering were valued by all case studies for their ability to expand the reach (numerically and geographically) of the KT strategy. Two case studies (*Cochrane Child Health* and *#ItDoesntHaveToHurt*) used specific social filtering techniques, utilizing social media influencers and hashtags to categorize messages, leading individuals to conversations and discussions pertaining to a specific topic or theme. These techniques also helped researchers tap into existing networks that amplified sharing by engaging partners and audiences through their own social media networks and interests.

One case study highlighted that the use of an expert media partner who could create compelling digital content and who already had an established reach ensured that content creation was done more efficiently and effectively than traditional academic-led dissemination campaigns. For other case studies, developing content was often time- and resource-intensive, and there was often a trade-off between effort and return. All case study leads agreed that having a team member or partner who specialized in digital content creation is likely an important element of a successful campaign (audience engagement and resource development).

#### Stakeholder Engagement

The case studies in which patient or parent partners co-designed and facilitated the strategies had an extended reach and higher level of engagement from the web community than the ones in which information was just pushed out by researchers. Capitalizing on a recognized and established network, community, or partnership enabled efficient and effective content creation and sharing.

The *Hirschsprung’s Disease Community* and *Parents Participating in Research Facebook Group* projects highlight that working with someone from the campaign’s intended audience (eg, parent and clinician) leads to relevant content being produced, developed, and created with that specific audience in mind.

Collaborating with a parent moderator who had credibility within the parent and caregiver community and who understood the needs of the researchers and the overall purpose of the collaborative helped nurture the interconnectedness between members of those web-based communities. It was proposed that having a moderator or stakeholder known within the community added to the perceived credibility of the campaign. Additionally, peer mediation was found to be a facilitating factor in the exposure and diffusion of information on the web. The *Parents Participating in Research Facebook Group* project found a shift in emphasis from its primary goal of creating an advisory group for the researchers to being an active group of knowledge exchange for parents (ie, asking questions, deciphering the research, and providing peer support). Parents reported feeling a sense of belongingness to the community where they could safely share stories, ask questions, and provide support for other members. This provided the researchers with a deeper understanding of what issues were most important to the daily lives of families, a unique insight that might not have come to fruition if the researchers were guiding the discourse.

#### Resource Requirements

Project teams and personnel needs varied across the case studies. Resources, types of skills, and tasks also varied within and between campaigns. Regardless of the campaign length, all projects required at least the equivalent of one full-time position. All case studies employed a research coordinator. Digital content creators, information specialists, students, or parent partners were also involved depending on the specific nature of the project.

### Evaluating

All case studies used various analytics (ie, Twitonomy, Facebook, Bitly, and Altmetric scores) to assess reach and engagement and understand how different facets of the strategies performed. A common evaluation component was web analytics to evaluate engagement (eg, likes, shares, and retweets) and uptake or use (eg, publication downloads and surveys completed) of information. However, not all web analytic programs were created equal. For example, harnessing accurate Facebook analytics with a private Facebook community was challenging because of privacy settings. Additionally, metrics such as Altmetric scores did not allow for the isolation of the study-specific impact and were time dependent (ie, older publications had more time to accumulate higher scores). The variability in the range of reporting periods for these statistics also influenced the usefulness of their collection, with availability ranging from a period of 30 days to all time. This was found to be a limiting factor when information was posted weekly or daily, making assessment of the impact of each post difficult.

A multimodal methodological approach (multiple qualitative and quantitative data collection) used by *#ItDoesntHaveToHurt*, *Hirschsprung’s Disease Community*, and the *Parents Participating in Research Facebook Group* helped evaluate less tangible aspects, such as passive involvement or interactions between community members or a shift from research teams pushing information out to audiences facilitating pull. However, it was felt that there was often a trade-off between scientific rigor (ensuring appropriate methods and analytics were used) and being able to adequately evaluate and report impact (more broadly than number of citations, downloads, etc). Case study authors noted that researchers need to understand that social media KT success is not an easily quantifiable measure and that unconventional methods of evaluating success may be needed.

A common challenge was the inability to assess and gain an understanding of passive involvement and attribute knowledge sharing to behavior change and its impact on health and health system outcomes. The main goals of most KT strategies were increasing awareness, knowledge, and uptake of evidence. Although analytics can provide details on proxy indicators (ie, reach, usefulness, use, and collaboration), the ability to assess more distal health outcomes was limited and perhaps unrealistic in these case studies.

### Ethics Considerations

The constraints placed by needing ethics approval to evaluate and capture participant behavior as well as to vet media posts (in some cases) was seen as a major challenge. One case study needed to have ethics approval for each social media post, which hindered their timeliness of engaging with the group on the web and highlighted a number of points for consideration. These include the value of spontaneous and organic conversation or the trade-off between not sharing information and the concern of providing health care advice rather than information to participants.

It was also noted that the need for ethics approval and informed participant consent was dictated by the outcomes evaluated. For example, the *Parents Participating in Research Facebook Group* set up rules of engagement that participants had to agree to before joining the community. However, to harvest the ideas and concerns of the group posted to the page to help other researchers know what was important to families, informed consent would have been needed. It felt that this may have stifled conversation, posing the following question: does the discourse of the web-based conversation change when participants are aware of ongoing monitoring and evaluation?

### Recommendations and Considerations

From these collective experiences, we put forward recommendations and points to consider when using social media as part of a KT strategy for health research. As with any KT initiative, key areas include clearly defining the purpose (ie, engagement, dissemination, and exchange or dialogue), goals, intended audience, type and volume of resources needed, project scope, study design, and evaluation. In addition, we encourage researchers to carefully consider the 3 explanatory and predictive components of social media (ie, posting frequency, social filtering, and negotiated awareness) when developing an approach and choosing a platform.

The checklist for researchers has been provided in Table 2.

**Table 2:** Checklist for researchers: what to ask and consider when developing a social media knowledge translation strategy.

Phase			Probing questions		Considerations
**Planning**					
	**Purpose and goal**				
		What is the purpose of using SM^a^ for this KT^b^ strategy?		Are you wanting to engage, disseminate, or create a dialogue?	
		What are the objectives of the campaign?		Identify a theory, framework, or model that aligns with the project’s purpose^c^ (eg, dissemination), objectives (eg, increase knowledge vs behavior change), and topic area (eg, health, sociology, and psychology)	
		N/A^d^		The scope of messages (ie, one clear message repeated over time in different formats or messages on multiple topics)	
		N/A		Create evaluation framework in planning phase	
		What will success look like?		What metrics for quantitative data will you collect and when (eg, at what point, how often, and for how long)?	
		How are you going to measure success?		Do you need qualitative evaluations?	
	**Intended audience**				
		Who is the intended audience?		Conduct formal or informal research to help answer these questions	
		What are the characteristics of the intended audience?		Conduct a social networking map of the intended audience	
		What SM platforms do they use? How do they interact on the web?		Involve some from the intended audience in planning (concepts of integrated KT)	
		How do they like to receive information?		This may differ for each end-user group	
	**Duration and intensity**				
		How long will the campaign run?		What resources do you have available?	
		How many posts a day or week or month?		This will be dependent on what platforms you use and your intended duration of interactivity	
		Can you incorporate social filtering?		What hashtags are currently used for your topic area, and is there already a presence on the web?	
**Doing**					
	**Platform and messaging**				
		What resonates with the intended audience?		Depending on resources and team expertise, you may use one or multiple platforms	
		Are they already gathered in an SM community or are you creating a new presence or community?		Invest in a SM management tool (eg, Hootsuite, Buffer, and Twitonomy)	
		What will work best for the campaign’s purpose?		Tailor strategy to ensure compatibility with each platform used	
		How will messages be structured and delivered (scope, format, plain language, figures, pictures, etc)?		Ensure messages are tailored to target audience needs	
		Are you incorporating social filtering or negotiated awareness techniques?		Map out who the influencers are that you want to reach	
		Have you reached out to stakeholders? Do you have a campaign champion (preferably a peer of the target group)?		Find someone who will act as a community or peer mediator to build trust within the target group	
	**Personnel, logistics, and other resources**				
		Who are the key personnel involved?		Content and communications experts are minimum requirements	
		What skill sets are needed?		Resources, time, and funding needed will differ across platforms	
		Do you have an advocate or opinion leader from the intended audience or community?		Connect with organizations with infrastructure already in place	
		How and how often will they post or interact with the audience or community?		N/A	
		Can you link in with a larger organization?		N/A	
	**Ethics**				
		Is everyone a research participant or peripherally involved because of interactions with a participant?		Evaluate the nature of participant involvement and prospectively consider the potential risks to individuals	
		Does the audience understand informed consent and expectations for privacy?		Tailor communication about risks and expectations	
		Are you aware of the privacy policies governing the different SM platforms?		Work closely with governing bodies and develop standards of practice (eg, create SM policy or “rules of engagement” document)	
**Evaluating**					
	**Planning**				
		Relates back to the goals—what are you hoping to achieve (eg, increased reach, engagement, and uptake)?		Create a logic model mapping goals to output indicators and ensure output indicators are measurable	
		How will you determine whether the SM campaign or strategy was successful?		Build an interim evaluation to ensure goals are being met and redeploy efforts and resources effectively	
	**Collecting**				
		Are you able to collect the data you need (eg, quantitative, qualitative, or both)?		Invest in web analytic tracking platforms	

^a^SM: social media.

^b^KT: knowledge translation.

^c^Useful resources: World Health Organization communications work [25], value-added research dissemination framework [26], and a guide to KT theory [27].

^d^Not applicable.

## Discussion

### Case Study Findings

Social media has become prevalent in many people’s lives as a means to communicate and exchange knowledge. The diverse formats of multiple, easily accessible platforms allow the creation, sharing, and exchange of ideas and information to various audiences. Users of social media can become interactive and are given equal opportunity for participation in the diffusion of information [28,29]. This shift in the communication landscape has paved the way for different stakeholders to interact and engage with intended end users in less traditional formats.

The case studies identified in this collection highlight the potential of using social media to engage with an intended audience and support collective action. Elements integral to the appeal of social media, personalization, presentation, and participation [30,31], overlap with those of KT. Furthermore, social media platforms may provide a rapid, accessible, and cost-effective means of implementing a widespread KT strategy [9]. Although there is no singular framework to guide the use of social media in a health research context, there are a number of factors that need to be considered when creating, implementing, and evaluating a social media KT strategy.

### Considerations

#### Understand Why You Want to Use Social Media as Part of Your Knowledge Translation Strategy

Social media has the power to engage with a wide variety of end users. Depending on the purpose of the strategy (eg, increase access to health information among the intended audience, increase reach and uptake of information, increase awareness or knowledge, and engage intended audience in dialogue [eg, for priority setting or information sharing]) and process (ie, letting it happen vs making it happen), there are many models that can be applied. These focus on dissemination, communication exchange, engagement, or behavior change [25,27,32-36].

We recommend that researchers select one or more social media platforms that align best with their purpose(s). For example, to create dialogue with patients who experience a common condition, a private Facebook group may be more appropriate than a live tweet chat. However, if the goal is broad dissemination, then tapping into an existing web network such as Twitter and posting frequently using social filtering and negotiated awareness techniques would be advantageous to rapidly reach a broader audience.

The scope of the messaging will also need to be taken into consideration during the development of the strategy. Focusing on a single message (one clear message repeated over time in different formats) or multiple messages (on multiple topics) will have implications for which platform you use, resourcing needs, and campaign length.

#### How to Understand and Engage Web Audiences?

Understanding your intended audience (eg, those engaged in a topic that can inform priority setting, those less engaged who need to access information, or those resistant to messaging and change in behavior) can help create a targeted strategy.

An in-depth understanding of the social network that you are trying to reach is also necessary to evaluate the current web environment. Understanding the structure of the web-based social networks of the intended audience might lead to the development of web-based algorithms that can detect trusted or influential users [37]. These users can then be identified and approached (in advance) to help engage a wider audience and disseminate information.

In the context of social media platforms, the users were self-selected; the question, therefore, becomes, how do we reach the users who were already there with the information that they need? Posting frequency is the grossest of metrics but can also be the most highly predictive [38-42]. Followers disengage when they perceive a channel or source to be dormant or dead. If you fail to post frequently in a noisy channel (eg, Twitter or any blog), you effectively do not exist. Additionally, people are more likely to draw close to those whose web activities are clear and apparent; this is why posting focused, tailored content frequently is beneficial. Users can then determine how the group of interest came to know of each other, what type of information (intent or agenda) they provide, and how it is being conveyed (personality). These factors can help them decide whether they want to connect with or follow the group. Social filtering, also collaborative filtering, is how a group organizes to allow people to find what could be useful or entertaining [43-45]. For example, if a tweet gets a lot of likes or retweets or a blog post is circulated across Twitter, Facebook, and numerous aggregators, this is interpreted as the judgment of the community.

By building on both direct (ie, proximate relationships and immediate relationships) and indirect (ie, distal relationships, connected by common factors, or influenced by external factors) social relationships, researchers can find users with similar interests. The impact this has on social capital (greater interactions generate a greater sense of community) should be considered and leveraged to enhance the success of a social media KT strategy. Targeted messaging and sharing content and knowledge that has been contributed or endorsed by other users can then be successfully capitalized on to reach the intended audience [46].

#### How Can a Social Media Platform Be Chosen?

Articulating the goal(s) of the strategy will help guide what platform should be used and for what purpose. Due to the unique functionality, interface, and content each platform offers, it is expected that people use and actively engage with the various platforms in different ways. For example, Voorveld et al [47] found that Facebook allowed people to respond, share, and be updated on information quickly (social interaction and topicality), whereas Twitter ensured people were quickly informed and up to date but had little topicality. Additionally, as there is often little overlap between different platforms regarding user demographics and conversations, multiple platforms may be necessary to reach all intended audiences [47,48].

Although empirical data to confirm whether cross-posting enhances reach and engagement are lacking, anecdotal evidence suggests that linking posts to multiple social media sites can enhance visibility and reach [47-49]. To gauge this, we suggest having representatives from the intended audience (eg, patient, consumer adviser, and other stakeholders) as part of the team or as a consultant. This will help determine which platform is most suitable and inform how best to engage with the intended audience from a messaging perspective. Additionally, whether one is establishing a presence on single or multiple channels, a pre-established platform or following is easier to partner with rather than building a new network during the campaign.

#### What Personnel and Resources Will You Require?

Many different skill sets and dedicated personnel are required to effectively execute a social media KT strategy [50]. Many grant-funded research projects are under resourced, and employing staff to come onboard for a select amount of time to execute a strategy may be unsustainable. To overcome some of the constraints placed on researchers, partnering with stakeholder organizations, networks, or advocacy units that have the necessary resources is advantageous. Additionally, media partners can alleviate some of the burden placed on research teams who do not have the necessary skill sets or time to develop multiplatform-compatible social media content or nurture a social media strategy to gain widespread traction.

Although Young et al [51] have suggested a framework for building an effective web-based health community through 4 key phases (ie, inception, establishment, maturity, and mitosis), the applicability of its use within an asynchronous or push-pull communication format via a single social media channel is yet to be determined. In other words, it seems feasible to create a closed Facebook group that is self-sustaining and that will grow organically, yet it is unclear if it is possible to create a similar model on a broader, less confined (less private) platform such as Twitter.

#### How Will You Evaluate Success and Impact?

The evaluation piece of a social media KT strategy needs to be embedded from the beginning (eg, to be able to see the number of times a link was accessed, the link needs to be set up as a traceable link before the strategy is implemented). There is an ongoing debate about which metrics are the most useful and relevant to determining the success of a KT strategy. Although the number of times a post is viewed or interacted with on social media may give an indication of reach, it is insufficient for assessing influence. Assessing the direct impact on change in attitude or behavior is not easily quantifiable, and alternative methods of measuring success are needed [52].

A range of quantitative analytics can be used to identify the components of a campaign that are most effective for assessing reach and uptake. However, consideration of their reporting periods and comparability across platforms is needed. If assessing reach and engagement, metrics such as likes, shares, and retweets are effective; traceable links and the number of downloads (often available from journals) are useful measures of engagement. Evaluating engagement in more depth can be done by posing questions or comments and assessing the reaction of the audience. However, unless these are monitored in real time, a retrospective evaluation of the number of comments and interactions between researchers and the consumer audience can be difficult and time-consuming (posts are often moved further down the conversation as more relevant posts are made). Although qualitative data collection could assist in this area, easily obtaining and collecting this information may not be feasible on a broad scale.

One construct that has the potential to enhance our understanding of KT success is social network analysis (SNA). SNA proposes a network-level perspective that examines how connections among individuals or entities and the nature of the associated interactions influence an outcome (eg, accessing or sharing evidence and changing practice behaviors based on evidence) [53]. However, little research evidence is available on the success of social media to improve the understanding or communication of health research findings to patients and families and the public (or those not specifically seeking treatment). Research in this area would benefit the health research community.

#### Do You Need Ethics Approval?

Regardless of whether evaluation is to be done formally or informally, the ethical considerations around web-based data collection and transmission need to be adhered to by researchers. As these are constantly evolving and changing to reflect both the researchers’ need for information and participants’ desire for privacy and confidentiality, it is imperative that research institutions and researchers are in agreement and compliance with what constitutes the need for informed consent in the digital world. As privacy policies are variable between social media sites and change frequently, they need to be checked before implementing a strategy, and the constraints imposed by varying privacy policies considered during the planning stages.

Ethics around the use of qualitative data acquired also need to be considered. Often, web-based patient groups can be a rich source of information for researchers wanting to understand the experience and views of patients around a particular condition or care need. The line between public and private domains then becomes blurred, with web-based data collection presenting unique issues during data transmission. According to Eysenbach and Wyatt [54], there are 3 main types of data collection and analysis possible from these web-based social media networks: (1) passive analysis, in which information posted on discussion groups is evaluated without researchers embedding themselves into the network; (2) active analysis, in which researchers participate in communications (response to questions by the group and posting questions to the group for discussion); (3) when researchers identify themselves and explicitly set out to gather information and data (surveys and focus groups). In the first 2 types, internet community members do not expect to be research subjects, which brings the need for and approach to consent into consideration.

Researchers and institutes need to work together to develop a code of conduct that benefits the participant as well as the project’s integrity and intended purpose. In 2015, the Connected and Open Research Ethics initiative was launched, providing practical and accessible guidance for researchers designing social media–enabled studies [55]. We recommend that researchers consult these guidelines during the development of any social media KT strategy.

### Limitations

Our study used a convenience sample of 4 pediatric case studies, which may not be representative of all health researchers’ experiences utilizing social media for KT. However, the generalist recommendations we provided are relevant to all health researchers and propose a broad set of considerations to others wanting to utilize social media for KT.

Although we did not specifically set out to complete a formal consensus building process (eg, Delphi), we acknowledge that this may have been a useful approach to avoid any bias associated with *group think*. Instead, we utilized deliberative dialogue. This process involved listening to other points of view; exploring and searching for new ideas, perspectives, and points of agreement; and bringing unexamined assumptions into the open. The end result produced a number of considerations based on collective insight and judgment on how best to utilize social media as part of a KT strategy.

As data were collected in 2017 to 2018, the findings may not reflect health researchers’ current experiences with social media. We evaluated a snapshot of social media use, which may have already changed given the direction and development of new platforms beyond Facebook and Twitter. The findings do, however, provide unique insights and ideas for future research.

### Conclusions

Social media has the potential to build and link web-based communities and to engage with, disseminate, and exchange information. Interconnections between people on social network sites as well as negotiated awareness and social filtering can enhance the process of information transfer and exchange and amplify the influence of that information. However, this comes with a cost. Ensuring adequate resources and time are available is essential to ensure a successful social media KT strategy.

A greater understanding of how best to evaluate social media as a KT tool for both active and passive engagement is needed to direct researchers in planning and evaluating their intended strategy. The elements and challenges discussed herein are important for researchers to consider to plan and evaluate a strategy that harnesses the power and personal aspects of social media for KT.

Perhaps a greater challenge is understanding how knowledge sharing and engagement contribute to behavior change and health outcomes and how we can gather and evaluate such outcomes. Further research is needed to help validate the proposed components and create a body of evidence around best practices for utilizing and evaluating social media as part of a KT strategy in health research.

## Appendix

Multimedia Appendix 1 Case study data collection form.

Multimedia Appendix 2 Case study descriptions.

Multimedia Appendix 3 Dimensions of communication.

## Abbreviations

KT: knowledge translation
SNA: social network analysis
SPOR: Strategy for Patient-Oriented Research
