# Supplementary material for: Considerations for Health Researchers Using Social Media for Knowledge Translation: Multiple Case Study
Source: J Med Internet Res. 2020 Jul 23;22(7):e15121. doi: 10.2196/15121 (PMC7413271; doi:10.2196/15121)
Supplement: Multimedia Appendix 1 [file jmir_v22i7e15121_app1.docx]

Title:

Topic:

Objectives:

End-user:

Project Logistics (Key components of social media strategy/campaign)

| **Intended audience** |  |
| --- | --- |
| **Social media platform(s) used** |  |
| **Intensity (frequency of posts etc)** |  |
| **Staffing requirements – FTE, # of Staff** |  |
| **Type of expertise used** |  |
| **Dimensions of communication used** |  |
| **Social filter used** |  |
| **Negotiated awareness used** |  |
| **Project timeline** |  |

Evaluation (What evaluation was done – what metrics were collected):

Key Findings:

Key Lessons Learned

| **What worked** | **Key challenges** |
| --- | --- |
|  |  |
|  |  |
|  |  |
|  |  |
|  |  |
