# Supplementary material for: Considerations for Health Researchers Using Social Media for Knowledge Translation: Multiple Case Study
Source: J Med Internet Res. 2020 Jul 23;22(7):e15121. doi: 10.2196/15121 (PMC7413271; doi:10.2196/15121)
Supplement: Multimedia Appendix 2 [file jmir_v22i7e15121_app2.docx]

### Box 1. Cochrane Child Health Case Study

The objective was to implement and evaluate a structured social media strategy, using multiple platforms, to disseminate Cochrane Child Health evidence to health care providers caring for children. The social media strategy had three components: daily "tweets" using the Cochrane Child Health Twitter account, weekly WordPress blog posts, and a monthly journal club on Twitter ("tweet chat"). Each tweet, blog, and journal club shared Cochrane evidence on a child health topic. The strategy was evaluated through (1) Twitter and blog site analytics, (2) traceable link (Bitly) statistics, (3) Altmetric.com scores for promoted evidence, and (4) participant feedback. Resources required to write the blog posts, tweet content, and manage the strategy were also recorded and evaluated.

The 22-week social media strategy ran between November 2014 and April 2015. Twenty-five blog posts were created, 585 tweets were posted, and three tweet chats were hosted over the course of the campaign. Monthly blog visits, views, and Twitter account followers increased over time. During the study period, the blog received 2555 visitors and 3967 page views from a geographically diverse audience of health care providers, academics, and health care organizations. In total, 183 traceable Bitly links received 3463 clicks and the Twitter account gained 469 new followers. Topics of interest varied within and between platforms. Clinical topics were the most visited and viewed blog posts, while popular topics on twitter were related to public health (vaccination) and pain management. We collected Altmetric.com scores for 61 studies promoted during the study period and recorded an average increase of 11 points. Research staff (n=3) contributed approximately 433 hours to promotion activities and planning (6.5 hours each per week) to implement the social media strategy, and study investigators reviewed all content (blog posts and tweets).

### Box 2. It Doesn’t Have to Hurt Case Study

The #ItDoesntHaveToHurt initiative aimed to share high quality, credible evidence in a parent-friendly manner and engage a panel of parents and partners. #ItDoesntHaveToHurt was a CIHR-funded social media initiative that spanned a 12-month period of targeted dissemination and discussion of content about children’s pain. All content was posted and promoted on the YummyMummyClub.ca website and through social media channels such as Twitter and Facebook. A mixed methods approach was used to evaluate reach and engagement of the #ItDoesntHaveToHurt content. Online analytics (e.g., # of views, likes, shares), online surveys and phone interviews with parents (conducted pre- and post-initiative) as well as formal partnership evaluation and media analysis were used to assess the reach and impact of the #ItDoesntHaveToHurt campaign.

The #ItDoesntHaveToHurt content had over 130 million content views over a 12-month period, and 4,570 instances of social media engagement. Earned media coverage on “key campaign topics” helped grow the success of the campaign by extending its reach. Parent surveys (n=2400) and interviews (n=200) showed significant increases in parent awareness and use of research evidence about children’s pain management.

### Box 3. Hirschsprung’s Disease Community: Prioritizing information gaps with parents of children with Hirschsprung’s Disease Case Study

The Hirschsprung’s Disease (HD) online community was developed in 2011 by a parent of a child with HD, to provide a forum for caregivers of children with HD to find and share information. The parent lead of this campaign works alongside a marketing team to develop, support and maintain the community. A partnership was developed between researchers and the parent lead to evaluate community needs and address information gaps.

The aim of the first parent-partnered study was to conduct a descriptive and quantitative analysis of the use of the social media community for HD [3]. Within this, a preliminary content analysis of priority areas for health information needs as identified by HD social media community members was conducted. Community responsiveness was assessed by quantifying the volume and timing of feedback for a question posted to Facebook by the site administrator. Results indicated that within 2 hours of posting, a question could receive 143 views and 20 responses, increasing to 30 responses after 5 hours. Priority areas for information needs were determined through a qualitative analysis to identify recurring themes within questions posed by members. Internet analytics including Google Analytics and Facebook Insights were used to evaluate overall the reach and responsiveness of the campaign. At the time of evaluation, the blog had been viewed over 5400 times from 37 countries; the Facebook page reached 46 countries with an average post reach of 298 users and 1414 “likes” and the campaign had 135 Twitter followers and 344 tweets.

A subsequent study engaged with the community members via an online survey to more formally assess information needs. Eighty-nine community members completed the survey; the majority of these were within the first week that the survey was posted. Results were merged with those from a survey of Canadian pediatric surgeons, and used to inform a review of the literature related to parent and surgeon-identified priorities [4] for information to guide the care of children with HD.

### Box 4. Establishment of a Parent-Led Web-Based Research Advisory Community for Childhood Disability Case Study

The goal of this community was to work together and exchange knowledge in order to improve research and the lives of children with disabilities and their families. This was supported through the development and evaluation of a Web-based research advisory community, hosted on Facebook and connecting a diverse group of parents of special needs children with researchers at CanChild Centre for Childhood Disability Research.

The Web-based Parents Participating in Research (PPR) advisory community was a secret Facebook group launched in June 2014 and run by two parent moderators who worked in consultation with CanChild. Its success was evaluated using Facebook statistics of engagement and activity (e.g., number of posts, number of comments) between June 2014 and April 2015. In addition, a web-based survey of members was used to evaluate the groups. The survey sought to ascertain information on several different aspects of the groups including descriptive demographics, research literacy levels, safety of the group, motivation for joining, perceived change from being a group member, and future direction of the group.

The PPR Facebook community had 96 participants (2 parent moderators, 13 researchers, and 81 family members) as of April 1, 2015. Over nine months, 432 original posts were made: 155 (35.9%) by moderators, 197 (45.6%) by parents and 80 (18.5%) by researchers. Posts had a median of three likes (range 0-24) and four comments (range 0-113). Members, rather than moderators, generated 64% (277/432) of posts. The survey had a 51% response rate (49/96 members), with 40 (82%) being parent members and nine (18%) being researchers. A total of 58% (23/40) of parents and 56% (5/9) of researchers indicated they felt safe to share sensitive or personal information. The initial purpose of the group was to be an advisory to CanChild, and 76% (28/37) of parents and all the researchers (9/9) identified having an impact on childhood disability research as their reason for participating.
