# Supplementary material for: Considerations for Health Researchers Using Social Media for Knowledge Translation: Multiple Case Study
Source: J Med Internet Res. 2020 Jul 23;22(7):e15121. doi: 10.2196/15121 (PMC7413271; doi:10.2196/15121)
Supplement: Multimedia Appendix 3 [file jmir_v22i7e15121_app3.docx]

# Appendix 1

## 1. Dimensions of Communication:

**Synchronous/Asynchronous**--Synchronous means collocated in time as a telephone call or face-to-face conversation. Asynchronous means not or not necessarily collocated in time as a play by Shakespeare published first as a folio 500 years ago.

**One to Many/Many to Many**--A printing press is one to many or at least fewer to many as a printing press requires a major capital investment, sophisticated division of labour. Same with a radio station. Many to many would be everyone can address everyone, as in online discussion fora or a graphitti wall.

**Static/Dynamic**--Static means that you cannot interact with it, like a written page or a stop sign. Dynamic means that you can interact with it, like the Amazon.ca site or filling out a tax form with a pen.

**Remote/Local**--Remote means that it arrives from a distance, as a broadcast signal or semaphore signals from another ship. Local means with you, in your face, like a live band in a club.

**Live/Recorded--**Live means happening now, in front of you, collocated in space. Recorded means that some media or medium is recreating a live moment, such as a cassette tape or an mp3.

**Ambient/Focal**--You push your cart through walmart; you hear muzak(tm) in the background. That's ambient, meaning it's all around you but not in your face. In a theatre everyone sits facing the stage or the screen, just as in a classroom. That would be focal, where there is one or more focal points to attend to.

**Push/Pull**--Push is media that knows your name as a letter addressed to you or an email message to you personally. Pull is media that tries to lure in you as websites online using Search Engine Optimization techniques (SEO) or books in a bookstore with their bright splashy covers and interesting titles.

**Durable/Ephemeral**--A book can last for a century or centuries, as can letter forms cut in stone. Not so vocalized syllables. Speech disappears even as you speak it.

**Phonetic-Syllabic-Logographic**--Phonetic scripts are those where letters resolve into sounds that resolve into meaning, like the Latin alphabet. Syllabic scripts are where letter forms resolve into syllables--search Awin writing. The Awin are a Javanese people. Logographic scripts are where letter forms stand for concepts, not speech, e.g. Chinese, Egyptian hieroglyphics, Ugurit Cuneiform. Logographic scripts often develop phonetic content over time.

**Iconic-Indexical-Symbolic**--Indexical signification means to point at something, as an arrow painted on the asphalt of an intersection can point to where traffic in a particular lane should go. Iconic means resemblance, as the stylized-silhouette leaping deer on a triangle sign warning you that deer may cross the road.
